# Chern and *Z*_2_ topological insulating phases in perovskite-derived 4*d* and 5*d* oxide buckled honeycomb lattices

**DOI:** 10.1038/s41598-019-53125-1

**Published:** 2019-11-21

**Authors:** Okan Köksal, Rossitza Pentcheva

**Affiliations:** 1 Department of Physics and Center for Nanointegration Duisburg-Essen (CENIDE), University of Duisburg-Essen, Lotharstr. 1, 47057 Duisburg, Germany; 2 Kavli Institute of Theoretical Physics, University of California at Santa Barbara, CA, 93106 USA

**Keywords:** Electronic properties and materials, Quantum Hall, Surfaces, interfaces and thin films, Topological insulators

## Abstract

Based on density functional theory calculations including a Coulomb repulsion parameter *U*, we explore the topological properties of (La*X*O_3_)_2_/(LaAlO_3_)_4_ (111) with *X* = 4*d* and 5*d* cations. The metastable ferromagnetic phases of LaTcO_3_ and LaPtO_3_ with preserved P321 symmetry emerge as Chern insulators (CI) with *C* = 2 and 1 and band gaps of 41 and 38 meV at the lateral lattice constant of LaAlO_3_, respectively. Berry curvatures, spin textures as well as edge states provide additional insight into the nature of the CI states. While for *X* = Tc the CI phase is further stabilized under tensile strain, for *X* = Pd and Pt a site disproportionation takes place when increasing the lateral lattice constant from *a*_LAO_ to *a*_LNO_. The CI phase of *X* = Pt shows a strong dependence on the Hubbard *U* parameter with sign reversal for higher values associated with the change of band gap opening mechanism. Parallels to the previously studied (*X*_2_O_3_)_1_/(Al_2_O_3_)_5_ (0001) honeycomb corundum layers are discussed. Additionally, non-magnetic systems with *X* = Mo and W are identified as potential candidates for *Z*_2_ topological insulators at *a*_LAO_ with band gaps of 26 and 60 meV, respectively. The computed edge states and *Z*_2_ invariants underpin the non-trivial topological properties.

## Introduction

Chern insulators and *Z*_2_ invariant topological insulators belong to subgroups of topological insulators (TIs) with and without broken time-reversal symmetry (TRS), respectively. A Chern insulator, also known as a quantum anomalous Hall insulator (QAHI) exhibits a quantized Hall conductivity without an external magnetic field^1,2^. In this context, CI are promising as potential candidates for the realization of Majorana fermions and the application in low-power electronics. The Chern insulator posseses chiral edge states with electrons traversing only in one direction, where the number of conducting edge states is determined by the Chern number^3^. A *Z*_2_ invariant TI supports the quantum spin Hall effect (QSHE) which can be regarded as two copies of an IQH (integer quantum Hall) system with electrons forming Kramers pairs and counter-propogating helical edge states. The properties of QSHE have been addressed in conjunction with the graphene lattice^4^ and HgTe quantum well structures^5–7^. Some further systems include stanene films functionalized with an organic ethynyl molecule (SnC_2_H)^8^, sandwiched 2D arsenene oxide (AsO) between boron nitride (BN) sheets^9^ and amidogen-functionalized Bi/Sb(111) films (SbNH_2_ and BiNH_2_)^10^. Lattices hosting a honeycomb pattern are of particular interest for topologically nontrivial states, as initially proposed by Haldane^11^. QAHI have been demonstrated in TIs doped with magnetic impurities such as Mn-doped HgTe or Cr-, Fe-doped Bi_2_Te_3_, Bi_2_Se_3_, Sb_2_Te_3_^12–14^. Another possibility to break TRS is by placing 5*d* transition metals on graphene^15,16^ or OsCl_3_^17^, as well as SnHN/SnOH^18^. Recently, transition metal oxides (TMO) have attracted interest due to their interplay of spin, orbital and lattice degrees of freedom. In contrast to conventional TIs whose bands near the Fermi energy are derived from *s* and *p*-type orbitals, the narrower *d*-bands lead to larger band gaps and a tendency towards TRS breaking. QAH phases have been predicted both for rocksalt- (EuO/CdO^19^ and EuO/GdO^20^), rutile-derived heterostructures^21–23^, pyrochlore oxides^24^ and 2D Nb_2_O_3_^25^. As noticed by Xiao *et al*.^26^, a buckled honeycomb lattice can be formed from two triangular *X*O_6_-layers in the *AX*O_3_ perovskite structure grown along the [111]-direction. Perovskite-derived bilayers of SrIrO_3_ and LaAuO_3_ were proposed as candidates for TIs, however interaction effects in the SrIrO_3_ bilayer lead to an AFM ground state^27,28^. 3*d* TM ions tend to host stronger electronic correlations and weaker spin-orbit coupling (SOC). Nevertheless, recently a strong SOC effect was encountered in (LaMnO_3_)_2_/(LaAlO_3_)_4_(111)^29^ which emerges as a Chern insulator with a band gap of 150 meV when the symmetry of the two sublattices is constrained. Since the ground state is a Jahn-Teller distorted trivial Mott insulator, selective excitation of phonons, as recently shown to induce an insulator-to-metal transition in NdNiO_3_/LaAlO_3_(001) SLs^30^, may present a pathway to suppress the symmetry breaking and access the CI state. Alternatively, 4*d* and 5*d* systems turn out to be less sensitive to symmetry breaking and the interplay of weaker correlation and stronger SOC makes them interesting candidates. This design principle served to identify LaRuO_3_ and LaOsO_3_ honeycomb bilayers sandwiched in LaAlO_3_(111) as Chern insulators^31^. Both Ru^3+^ (4*d*^5^) and Os^3+^ (5*d*^5^) are in the low-spin state with a single hole in the *t*_2*g*_ manifold whereas the homologous Fe^3+^ (3*d*^5^) in LaFeO_3_ is found to be in a high-spin state with an AFM ground state.

**Figure 1:**
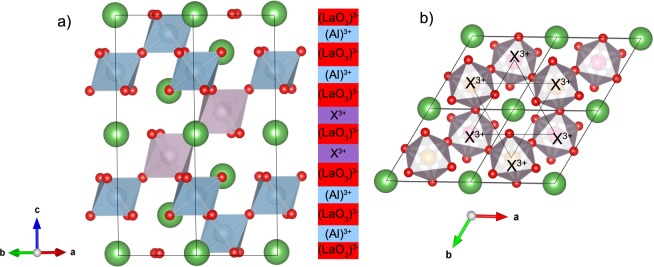
(**a**) Side view of the (La*X*O_3_)_2_/(LaAlO_3_)_4_ (111) superlattice where *X* represents a 4*d* or 5*d* cation. (**b**) Top view of the buckled honeycomb lattice in the *a*-*b* plane illustrating the corner-sharing octahedra in the perovskite whereas solid and black lines connect the next nearest TM-ion neighbors residing on the same sublattice.

A honeycomb pattern arises also in the corundum structure, albeit with smaller buckling and different connectivity. While in the perovskite structure the octahedra are corner sharing (cf. Fig. 1), in the corundum-derived SLs the *X*O_6_ octahedra in the *X*_2_O_3_ layer are edge-sharing as well as alternating corner- and face-sharing to the next layer above and below. The complex electronic behavior of corundum-derived honeycomb layers (*X*_2_O_3_)_1_/(Al_2_O_3_)_5_ (0001) was recently addressed in a systematic study of the 3*d* series^32^. Moreover, among the 4*d* and 5*d* systems the ferromagnetic cases of *X* = Tc, Pt were identified as Chern insulators with *C* = −2 and −1 and band gaps of 54 and 59 meV, respectively^33^. This motivated us to explore here the perovskite analogues *X* = Tc, Pd, and Pt in (111)-oriented (La*X*O_3_)_2_/(LaAlO_3_)_4_. Although the ground state is AFM, we find that a CI phase emerges for the metastable FM cases with *C* = 2 and 1. Furthermore, we explore the effect of strain on the stability of the CI state, as well as the dependence on the Coulomb repulsion parameter *U* and compare to the corundum-type systems. Last but not least, we concentrate on TI cases where time reversal and inversion symmetry are preserved and identify the non-magnetic phases of *X* = Mo, W in (La*X*O_3_)_2_/(LaAlO_3_)_4_ (111) superlattices as potential candidates for *Z*_2_ TIs.

## Theoretical Methods

Density functional theory calculations were performed for (La*X*O_3_)_2_/(LaAlO_3_)_4_ (111) SLs employing the projector augmented wave (PAW) method^34^ as implemented in the VASP^35^ code. The plane-wave cutoff energy is fixed to 600 eV. The generalized gradient approximation (GGA) in the parametrization of Perdew-Burke-Enzerhof  ^36^ was used for the exchange-correlation functional. The static local correlation effects were accounted for in the GGA + *U* approach, using ${U}_{{\rm{eff}}}=U-J$ of Dudarev *et al*.^37^. Hubbard *U* values for the 4*d* and 5*d* ions are typically lower than for the 3*d* cations^31,33^. We used an *U* = 3 eV for *X* = Tc, Pd, Mo and 1–2 eV for *X* = Pt, W and a Hund’s exchange parameter of *J* = 0.5 eV in all cases. Additionally, *U* = 8 eV is used for the empty 4 *f* orbitals of La. The calculations were performed using a $\Gamma$ centered *k*-point grid of 12 × 12 × 2. The lattice parameter *c* and the internal coordinates of the superlattice structure were optimized until the Hellman-Feynman forces were less than 1 meV/Å. SOC was considered in the second-variational method with magnetization along the (001) quantization axis. For potentially interesting cases maximally localized Wannier functions (MLWFs) were constructed in order to calculate the Berry curvatures and the anomalous Hall conductivity (AHC) on a dense *k*-point mesh of 144 × 144 × 12 using the wannier90 code^38^.

## Results and Discussion

Our previous study^33^ on corundum-derived superlattices with honeycomb pattern and  $X=4d$ or 5*d* ion showed that the metastable ferromagnetic cases of *X* = Tc, Pt host quantum anomalous Hall states. Using the insight gained from this investigation, we explore the perovskite-derived SLs with the above-mentioned TM ions. Although the ground states of *X* = Tc, Pd and Pt in (La*X*O_3_)_2_/(LaAlO_3_)_4_ (111) superlattices are AFM, (cf. Table 1) with symmetry lowering due to a dimerization, manifested in alternating *X*–*X* bond lengths (not shown here), we concentrate here on the metastable ferromagnetic phases and explore their topological properties. Moreover, we investigate the effect of strain on the Chern insulating phases by considering two in-plane lattice constants of substrates LaAlO_3_ (3.79 Å) and LaNiO_3_ (3.86 Å) which corresponds to a change in strain of ~1.8%. We futhermore extend our study to non-magnetic solutions leading to *Z*_2_ TIs. In particular, *X* = Mo, W turn out to possess *Z*_2_ topologically invariant phases, albeit their non-magnetic phases are higher in energy by 2.0 eV and 0.4 eV per u.c., respectively, compared to the antiferromagnetic ground states (cf. Table 2).

**Table 1:** Structural, electronic and magnetic properties of the FM state in *X* = Tc, Pt, Pd SLs at *a*_LAO_ and *a*_LNO_, respectively.

LaTcO_3_	*a*_LAO_	*a*_LNO_
*c*[Å]	13.9	13.8
$\Delta {E}_{{\rm{FM}}-{\rm{AFM}}}$[eV]	1.0	—
*M*_*S*_[*μ*_B_]	1.96/1.96	1.96/1.96
*M*_*L*_[*μ*_B_]	0.06/0.06	0.06/0.06
E_*g*_[meV]	m	m
E_*g*(SOC)_[meV]	41	53
*C*	2	2
LaPdO_3_	*a*_LAO_	*a*_LNO_
***c***[Å]	14.0	13.7
$\Delta {E}_{{\rm{FM}}-{\rm{AFM}}}$ [eV]	0.8	—
*M*_*S*_[*μ*_B_]	0.71/0.72	0.60/0.85
*M*_*L*_[*μ*_B_]	0.05/0.05	0.05/0.07
E_*g*_[meV]	m	60
E_*g*(SOC)_[meV]	m	70
*C*	0	0
LaPtO_3_	*a*_LAO_	*a*_LNO_
***c***[Å]	14.2	14.1
$\Delta {E}_{{\rm{FM}}-{\rm{AFM}}}$[eV]	1.1	—
*M*_*S*_[*μ*_B_]	0.67/0.67	1.06/0.32
*M*_*L*_[*μ*_B_]	0.13/0.13	0.25/0.05
E_*g*_[meV]	m	370
E_*g*(SOC)_[meV]	38	402
*C*	1	0

The relative energy difference of ferromagnetic (FM) configuration with respect to the antiferromagnetic (AFM) ground state, spin and orbital moments, band gaps with and without SOC (“*m*” denotes a metallic state) and Chern numbers are also listed.

**Table 2:** Structural, electronic and magnetic properties of *X* = Mo, W SLs in (La*X*O_3_)_2_/(LaAlO_3_)_4_ (111).

	LaMoO_3_	LaWO_3_
	*a*_LAO_	*a*_LAO_
*c*[Å]	14.1	14.2
$\Delta {E}_{{\rm{NM}}-{\rm{AFM}}}$[eV]	2.0	0.4
E_*g*_[meV]	26	67
E_*g*(SOC)_[meV]	26	60
*Z*_2_	1	1

The relative energy difference of the non-magnetic (NM) configuration with respect to the antiferromagnetic (AFM) ground state, band gaps with and without SOC and Z_2_ indices are listed.

### GGA + *U* ( + SOC) results: *X* = Tc

**Figure 2:**
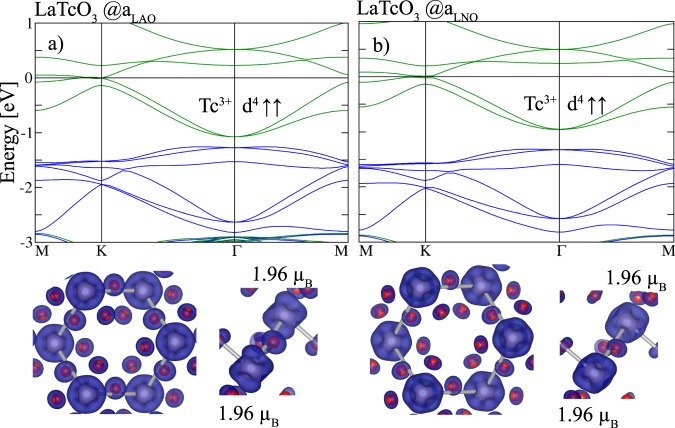
Spin-resolved band structure of the buckled bilayers for *X* = Tc with corresponding lattice constants (**a**) *a*_LAO_ and (**b**) *a*_LNO_, respectively. In the band structures blue/green denote majority/minority bands and the Fermi level is set to zero. The lower two panels depict the top and side view of isosurfaces of the spin densities in the integration range of −3 eV to *E*_F_ where blue (red) show the majority (minority) contributions.

In the following, we discuss the electronic and topological properties of ferromagnetic *X* = Tc for in-plane lattice constants *a*_LAO_ and *a*_LNO_. Without SOC and for *U*_eff_ = 2.5 eV semi-metallic band structures emerge as depicted in Fig. 2a,b. In both cases the band structures around *E*_F_ are very similar and dominated by minority Tc *t*_2*g*_ bands (cf. Fig. 2a,b) touching at K, that extend to ~−1.4 eV (*a*_LAO_) and ~−1.0 eV (*a*_LNO_) and are completely separated from the lower lying majority bands. This feature is dissimilar to the band structure in the corundum honeycomb layer (Tc_2_O_3_)_1_/(Al_2_O_3_)_5_(0001)^33^ where the majority and minority bands are entangled around *E*_F_.

The spin densities are shown in Fig. 2a and b. In the Tc^3+^ 4*d*^4^ configuration all electrons are in the *t*_2*g*_ subset with two unpaired electrons, reflected in a magnetic moment of 1.96 *μ*_B_ for both Tc sites. This is in contrast to the corundum honeycomb layer with *X* = Tc^33^ where a much lower magnetic moment of 0.93 *μ*_B_ is found resulting from a violation of Hund’s rule due to a strong hybridization between Tc 4*d* and O 2*p* states.

**Figure 3:**
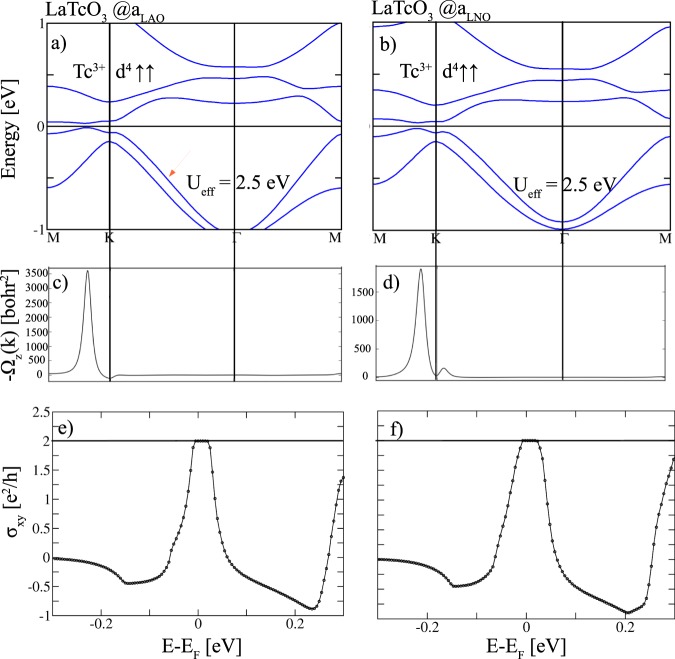
GGA + *U* + SOC band structures for *X* = Tc in (111)-oriented perovskite bilayer at (**a**) *a*_LAO_ and (**b**) *a*_LNO_. The Berry curvatures (**c**,**d**) are plotted along the same *k*-path and (**e**,**f**) show the corresponding anomalous Hall conductivities *σ*_*xy*_ vs. the chemical potential in units of ${e}^{2}/h$.

We proceed with the effect of SOC on the band structures illustrated in Fig. 3a,b. The strongest influence is observed at the Fermi level around the K point where SOC induces anti-crossings and opens gaps of 41 and 53 meV for *a*_LAO_ and *a*_LNO_, respectively. Calculation of the AHC (Fig. 3e,f) shows that the LaTcO_3_ honeycomb layer emerges as a Chern insulator with *C* = 2 for both strain values. The largest contributions to the Berry curvature $\Omega (k)$ (Fig. 3c,d) arises along K-M. The enhanced gap for *a*_LNO_ leads to a broader Hall plateau at *E*_F_ (cf Fig. 3e,f). The results demonstrate that the CI phase is further stabilized under tensile strain. A similar effect of strain was observed in (Tc_2_O_3_)_1_/(Al_2_O_3_)_5_(0001)^33^. We note that the sign of the Chern number *C* = 2 for *X* = Tc in the (111)-oriented perovskite bilayer is reversed compared to the corundum-derived SL (*C* = −2)^33^. The reversal of sign is related to the specific band topology and band gap opening mechanism and the predominance of minority bands, whereas in the corundum case majority bands reside around *E*_F_^33^.

### GGA + *U* results for isoelectronic *X* = Pd, Pt

**Figure 4:**
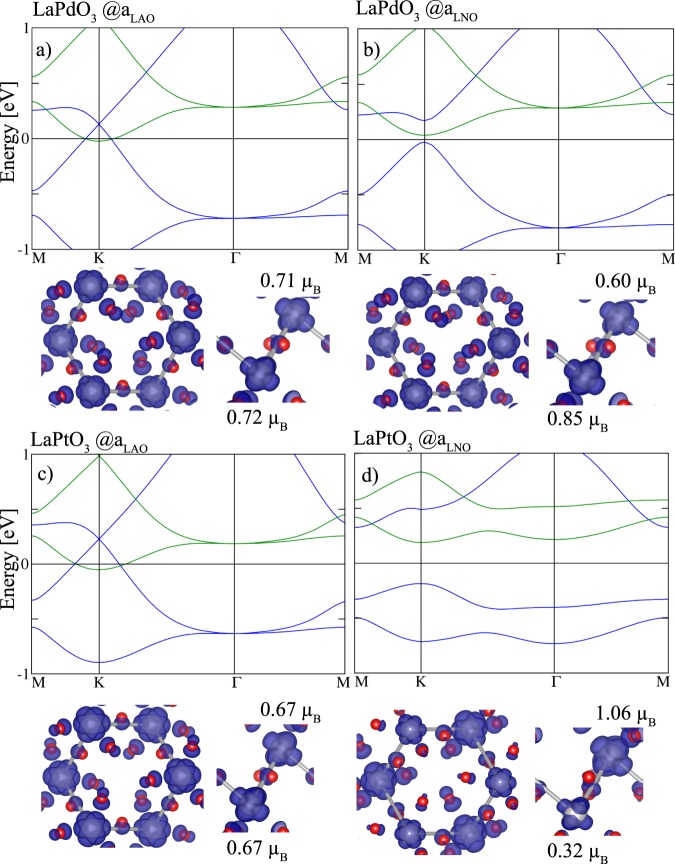
Spin-resolved band structure of (111)-oriented (La*X*O_3_)_2_/(LaAlO_3_)_4_ perovskite bilayer (**a**–**c**) at *a*_LAO_ and (**b**–**d**) *a*_LNO_ for the isovalent and isoelectronic *X* = Pd and Pt at *U*_eff_ = 2.5 eV and 1.0 eV. The top and side view of isosurfaces of the spin densities are integrated in the energy range between −1 (Pt) and −1.2 eV (Pd) and *E*_F_ where blue (red) show the majority (minority) contributions.

We next turn to the isoelectronic *X* = Pd and Pt. Experimental studies suggest paramagnetic metallic behavior for bulk LaPdO_3_^39,40^. For the honeycomb layer of *X* = Pd and Pt at *a*_LAO_ *P*321 symmetry is preserved and the band structures in Fig. 4a,c show two very similar sets of four majority and minority bands, the former lying about 1 eV lower than the latter. Both exhibit Dirac crossings at K, the one of the majority band being slightly above the Fermi level. Consequently, the dispersive majority and the bottom of the minority bands cross *E*_F_ and lead to a metallic state. This differs from the isoelectronic LaNiO_3_ analogon where for *P*321 symmetry the Dirac point is fixed at the Fermi level^29,41–43^. A substantial occupation of both *e*_*g*_ orbitals and contribution from the O 2*p* states is visible from the spin-densities (see Fig. 4) which indicates a *d*^8^*L* configuration instead of the formal *d*^7^ occupation and bears analogies to the isoelectronic LaNiO_3_^43–45^. In contrast, for tensile strain (*a*_LNO_) the P321 symmetry is lowered and a gap of ~60 meV and ~370 meV (cf. Fig. 4b,d) is opened for LaPdO_3_ and LaPtO_3_, respectively. The gap opening arises due to the disproportionation of the two Pd and Pt triangular sublattices expressed in different magnetic moments: In *X* = Pd the two sites acquire magnetic moments of 0.85 *μ*_B_ and 0.60 ${\mu }_{{\rm{B}}}$ (cf. Fig. 4b and Table 1). For *X* = Pt this site-disproportionation is more pronounced with magnetic moments of 1.06 ${\mu }_{{\rm{B}}}$ and 0.32 ${\mu }_{{\rm{B}}}$ on the two Pt sites (cf. Fig. 4d) resulting in a larger gap between the occupied majority and unoccupied minority pairs of bands whose dispersion is significantly reduced. The site-disporportionation at tensile strain goes hand in hand with a breathing mode expressed in a larger and a smaller PtO_6_ octahedron with volumes of 13.8 and 11.8 Å^3^, respectively. As a consequence, the Pt-O bond lengths result in 2.17, 2.18 Å at the first and 2.03, 2.10 Å at the second Pt site. Such a disproportionation is common in bulk rare earth nickelates^46,47^, (001) or (111)-oriented LaNiO_3_/LaAlO_3_ SLs^29,43–45,48,49^ as well as La_2_CuO_4_/LaNiO_3_(001) SLs^50^.

### *X* = Pt: Emergence of a CI phase as a function of *U*

**Figure 5:**
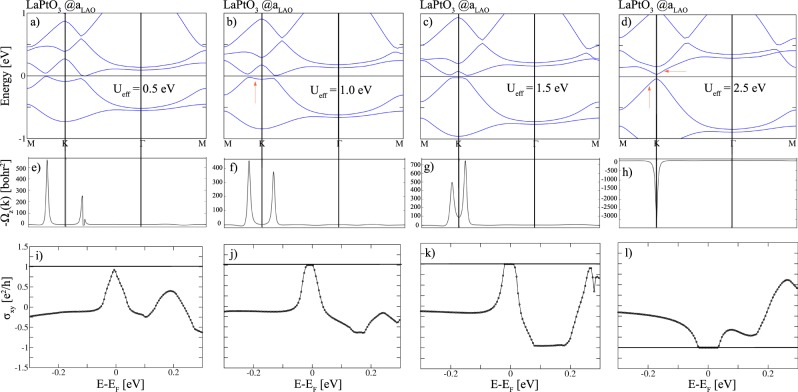
GGA + *U* + SOC results for (111)-oriented (LaPtO_3_)_2_/(LaAlO_3_)_4_ as a function of the Coulomb repulsion parameter *U*: Evolution of the band structure (**a**–**d**), Berry curvatures (**e**–**h**) plotted along the same *k*-path and AHC *σ*_*xy*_ vs. the chemical potential (**i**–**l**).

Since at *a*_LNO_ both LaPdO_3_ and LaPtO_3_ result in trivial Mott insulators due to site disproportionation, we explore here the topological properties at *a*_LAO_, where the P321 symmetry is preserved. Upon including SOC, for *X* = Pd a CI phase emerges for *U* values beyond ${U}_{{\rm{eff}}}^{{\rm{c}}}=3.5\,{\rm{eV}}$ (not shown here), which are likely too high for a 4*d* element. We concentrate here on the topological properties of (LaPtO_3_)_2_/(LaAlO_3_)_4_(111) as a function of Hubbard *U*. Up to *U*_eff_ = 2.0 eV SOC leads to a band inversion between the majority and minority bands around K. At *U*_eff_ = 0.5 eV the conduction band still overlaps with the Fermi level (cf. Fig. 5a) and hampers the formation of a quantized Hall plateau (see Fig. 5i). For 1.0 < *U*_eff_ < 2.0 eV (cf. Fig. 5b,c), the Fermi level lies inside the gap arising from band inversion between bands of opposite spin and the system becomes a Chern insulator with *C* = 1. Increasing the Coulomb repulsion strength from 1.0 to 1.5 eV enhances the band gap (from 31 to 38 meV) and the Hall plateau which stabilizes the Chern insulating phase (see Fig. 5k). As can be seen from Fig. 5f,g positive contributions to the Berry curvature arise around K. For higher values (*U*_eff_ ≥ 2.5 eV) the effect of SOC changes from band inversion between bands of opposite spin to avoided crossing between two bands with the same spin with a larger gap of 66 meV, leading to a sign reversal of the Chern number from + 1 to −1 (cf. Fig. 5l). This is consistent with the large negative Berry curvature contribution around K in Fig. 5h. While for 5*d* systems *U* values beyond 2.0 eV appear to be too high, we note that the band structure for ${U}_{{\rm{eff}}}=2.5\,{\rm{eV}}$ is similar to the results from a calculation with the hybrid functional HSE06^51,52^ with standard mixing parameter of exact exchange of 0.25 (See Supplemental Material for additional information on electronic and structural properties).

### Edge states and spin textures of CI phases

**Figure 6:**
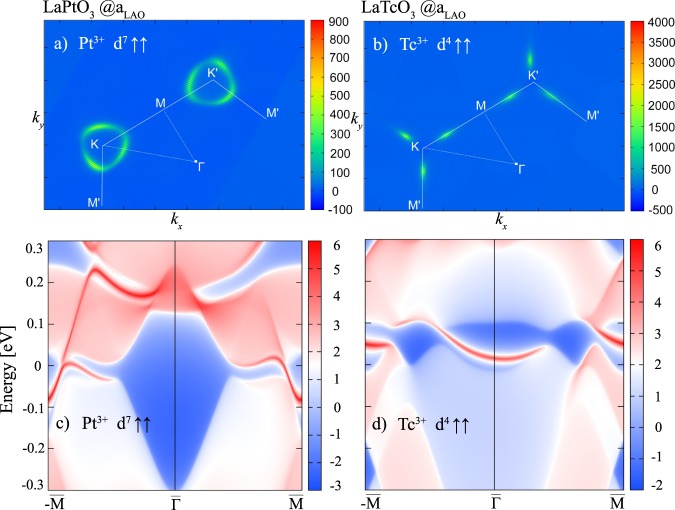
Top view of the Berry curvatures $\Omega (k)$ for (**a**) *X* = Pt (**b**) Tc in (111)-oriented (La*X*O_3_)_2_/(LaAlO_3_)_4_ at *U*_eff_ = 1.0 eV and 2.5 eV in the Chern-insulating phase. The calculated edge states *X* = Pt, Tc superlattices are shown in (**c**,**d**) for (100) surfaces. Red-white range of colors represent higher local DOS, the solid red lines correspond to the edge states connecting valence and conduction bands. The blue regions denote the bulk energy gap. The Fermi level is set to zero.

Unlike in (Pt_2_O_3_)_1_/(Al_2_O_3_)_5_(0001)^33^ where contributions to the Berry curvature $\Omega (k)$ arise along M and K, the top view of the Berry curvature for *U*_eff_ = 1.0 eV (see Fig. 6a) reveals that non-vanishing contributions appear solely on a rounded triangular feature around K marking the anticrossing line of the majority and minority band. The surface state in Fig. 6c calculated employing the MLWF method^53^ presents a single chiral edge state associated with *C* = 1. For *X* = Tc the largest contribution to $\Omega (k)$ emerges along K-M (cf. Fig. 6b,d) resulting in two in-gap chiral states whose features are similar to (Tc_2_O_3_)_1_/(Al_2_O_3_)_5_(0001)^33^.

**Figure 7:**
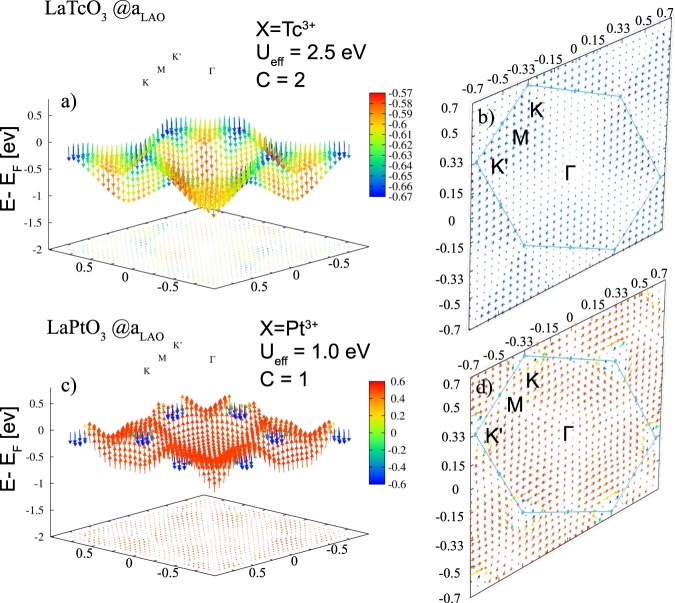
Side and top view of the spin textures in *k*-space from GGA + *U* + SOC calculations with out-of-plane magnetization of the highest occupied bands for (**a**,**b**) *X* = Tc (cf. Fig. 3a) at *U*_eff_ = 2.5 eV and (**c**,**d**) for *X* = Pt (cf. Fig. 5b) at *U*_eff_ = 1.0 eV. The color scale provides the projection of the texture field on the $\hat{z}$-axis with red (blue) indicating parallel (antiparallel) orientation for *X* = Pt. In contrast, for *X* = Tc only negative values with varying size are observed. The top views display the in-plane variation of the spins.

Here we briefly address the spin texture of the highest occupied band (marked by arrows in Figs 3a and 5b) in the Chern insulating phase for *X* = Tc and *X* = Pt, respectively. For *X* = Pt (see Fig. 7c) the spin texture is dominated by majority (red) components in the larger part of the BZ and exhibits an orientation reversal of minority (blue) *s*_*z*_ spin components close to K, consistent with the SOC-induced band inversion between the occupied majority and unoccupied minority band around K discussed above. The spin texture of (LaTcO_3_)_2_/(LaAlO_3_)_4_(111) in Fig. 7a is rather collinear and exhibits only negative *s*_*z*_ values throughout the entire BZ. This is consistent with the fact that only bands of minority character appear around *E*_F_. Overall, even though the number of edge states of the perovskite and corundum case (Tc_2_O_3_)_1_/(Al_2_O_3_)_5_(0001)^33^ are identical, the differences (we remind that in the corundum case a vortex arises around $\Gamma$) can be attributed to the distinct electronic structure and the effect of SOC, as discussed in Section III A.

### *Z*_2_ topological invariant systems: GGA + *U* ( + SOC) results for isoelectronic *X* = Mo, W

**Figure 8:**
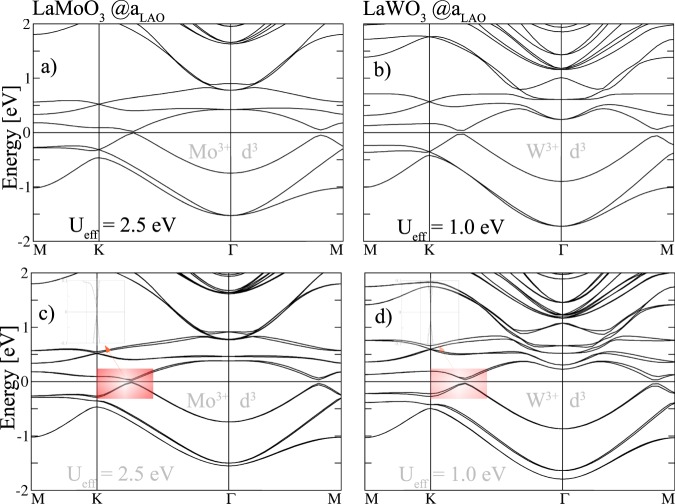
Non-magnetic band structures without (**a**,**b**) and with SOC (**c**,**d**) of the buckled bilayers *X* = Mo and W for *U*_eff_ = 2.5 eV and 1.0 eV at the in-plane lattice constant *a*_LAO_, respectively. The Fermi level is set to zero.

Besides the potential CI phases in *d*^7^ and *d*^4^ systems studied above, we investigate the non-magnetic phases of the two *d*^3^ systems containing the homologous elements *X* = Mo and W at *a*_LAO_. Despite the AFM ground state (cf. Table 2), the potentially interesting systems were identified to be non-magnetic. We note that previous theoretical studies^54^ suggest that bulk LaMoO_3_ and LaWO_3_ should be non-magnetic. The band structures in Fig. 8a,b reveal that the bandwidth of the *t*_2*g*_ manifold amounts to ~1.8 eV for *X* = W and ~1.5 eV for *X* = Mo. The larger bandwidth correlates with the larger extension of the 5*d* orbitals as compared to 4*d*. In contrast to bulk LaMoO_3_ and LaWO_3_^55^ which are metallic, the non-magnetic perovskite superlattices exhibit semiconducting behavior with gaps of 28 meV and 62 meV, that are only weakly modified by SOC, 26 meV and 60 meV, respectively. Nevertheless, the degeneracy of bands is lifted along K-$\Gamma$ (cf. Fig. 8c,d).

**Figure 9:**
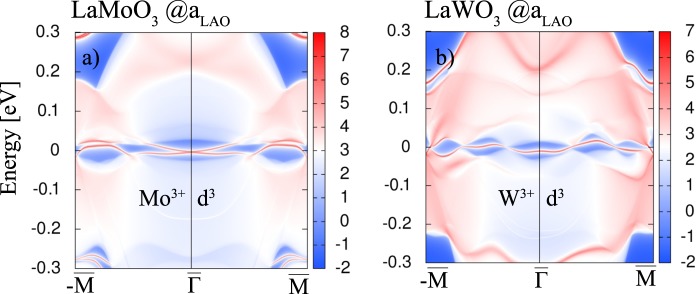
The edge states of *X* = Mo, W superlattices shown in (**a**,**b**) for (100) surfaces with same color coding as in Fig. 6.

**Figure 10:**
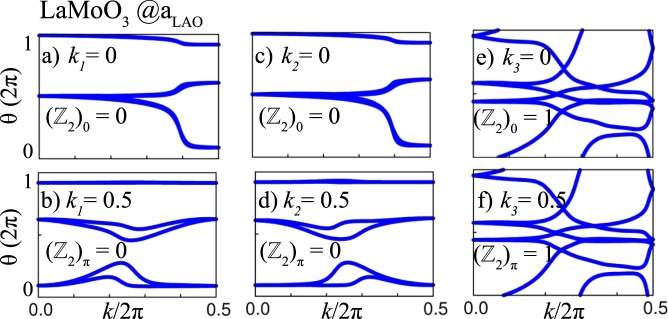
The evolution of the Wannier charge centers (WCCs) on six time-reversal invariant momentum planes shown in (**a**–**f**) for *X* = Mo.

In order to verify the topological features of these two systems, we carry out edge state calculations by constructing the MLWFs. The edge Green’s function and the local density of states (LDOS) can be simulated using an iterative method^53,56,57^. In the case of *X* = Mo, one can clearly see a gapless Dirac cone at the $\Gamma$ point (cf. Fig. 9a), whereas one topologically protected chiral edge state is obtained for *X* = W (cf. Fig. 9b) connecting the valence and conduction bands. We note that additional background LDOS may arise owing to hybridization with trivial edge states emerging due to the particular procedure of creating the edge interfaced with vacuum. Similar effects have been observed previously^9,25,58^. Since the investigated systems have crystal IS and TRS, *Z*_2_ can be calculated as a product of parities of all occupied states at the TRIM points by applying the criterion of Fu and Kane^59^. In Fig. 10a–f the Wannier function center evolution (WCC) is calculated for *X* = Mo using the Wilson loop method^60,61^. For the *k*_1_ and *k*_2_ planes the *Z*_2_ indices are 0 whereas for *k*_3_ = 0 and *k*_3_ = 0.5 the *Z*_2_ indices yield 1.

## Summary

In summary, we investigated the possibility to realise topologically nontrivial states in (111)-oriented perovskite-derived honeycomb La*X*O_3_ layers with *X* = 4*d* and 5*d*, separated by the band insulator LaAlO_3_. The metastable ferromagnetic phases of (LaTcO_3_)_2_/(LaAlO_3_)_4_(111) and (LaPtO_3_)_2_/(LaAlO_3_)_4_(111) emerge as CI with *C* = 2 and 1, respectively, at the lateral lattice constant of LaAlO_3_ (3.79 Å). Thereby, the persistence of P321 symmetry, lattice strain and the inclusion of a realistic Hubbard *U* term turn out to be crucial. For *X* = Pd the CI phase appears for *U* beyond 3.5 eV that likely exceeds the realistic range for a 4*d* compound. For *X* = Pt the sign of the Chern number is reversed beyond *U*_eff_ = 2.0 eV due to change of the spin orientation of the contributing bands. The CI phase for (LaTcO_3_)_2_/(LaAlO_3_)_4_(111) is further stabilized under tensile strain (at the lateral lattice constant of LaNiO_3_), similar to the corundum-based SL (Tc_2_O_3_)_1_/(Al_2_O_3_)_5_(0001). In contrast, for *X* = Pd and Pt in (La*X*O_3_)_2_/(LaAlO_3_)_4_ (111) tensile strain lifts the P321 symmetry and induces a site-disproportionation on the two sublattices which opens a trivial band gap and bears analogies to the behavior of the isoelectronic nickelate superlattices^29,43–45,48,49^. Further insight into the topological aspects is gained by analyzing the Berry curvatures, edge states and spin textures. A closer inspection of the spin texture for LaPtO_3_ reveals a spin orientation reversal along the loop of band inversion around K of two bands with opposite spin character. Moreover, we explored non-magnetic perovskite SLs where TRS is preserved and identified *X* = Mo and W as potential candidates for *Z*_2_ TIs. The existence of edge states and non-trivial *Z*_2_ indices supports this outcome.

We note that most of the effects (i.e. emergence of topologically nontrivial (Chern) insulating phases) we observe are interaction-driven, i.e. they do not appear within DFT/GGA (*U* = 0 eV) calculations. On the other hand with increasing static correlation effects also the tendency towards stabilization of trivial Mott insulating phases connected with symmetry lowering is enhanced. This applies not only to the systems we address here which show AFM ground states but has been observed in several previous studies^28,32,33^. Although the specific systems proposed here have not yet been synthesized, recent experimental studies reported the successful growth of related (111)-oriented nickelate superlattices^62–64^ as well as nickelate and manganate perovskite heterostructures^65^. Thus, we trust that our theoretical predictions will encourage further experimental efforts to realize and characterize the proposed systems.

## Supplementary information

Supplemental material
