# Supplementary material for: Chern and Z2 topological insulating phases in perovskite-derived 4d and 5d oxide buckled honeycomb lattices
Source: Sci Rep. 2019 Nov 21;9:17306. doi: 10.1038/s41598-019-53125-1 (PMC6872743; doi:10.1038/s41598-019-53125-1)
Supplement: Supplementary file 1 — Supplemental material [file 41598_2019_53125_MOESM1_ESM.pdf]

Supplemental material: Chern and Z_2 topological insulating phases in perovskite-derived $4d$ and $5d$ oxide buckled honeycomb lattices

Okan Köksal[1] and Rossitza Pentcheva[1,*]

[1] *Department of Physics and Center for Nanointegration Duisburg-Essen (CENIDE), University of Duisburg-Essen, Lotharstr. 1, 47057 Duisburg, Germany*

(Dated: September 28, 2019)

ELECTRONIC PROPERTIES

For the $(\mathrm{La}X\mathrm{O}_3)_2/(\mathrm{LaAlO}_3)_4$ perovskite bilayers oriented along [111]-direction at a_{LAO} for $X = \mathrm{Tc}$ and Pt, we have performed HSE06 calculations[1, 2]. As the band structures in Fig. 1 reveal, the main features of the band structure for $X = \mathrm{Tc}$ (cf. Fig. 1a) are reproduced (see Fig. 2a in the main manuscript) also for the hybrid functional calculations with only a slight shift above E_{F} for the minority Tc t_{2g} bands at K. For$X = \mathrm{Pt}$ (cf. Fig. 1b) the majority bands exhibit Dirac crossings at K $\sim$ 0.5 eV above E_{F} whereas for the unoccupied minority bands the shift is more pronounced lying $\sim$ 1 eV above E_{F}. In this case the band structure corresponds rather to the GGA+U result for a higher U value of 2.5 eV (cf. Fig. 5d in the main manuscript). We point out that hybrid functionals also contain a mixing parameter α, that renders the fraction of exact exchange. There are many studies where values different from the standard value of 0.25 are chosen, sometimes determined in a self-consistent fitting procedure to the dielectric constant [3]. This goes however beyond the scope of the present study. Nevertheless, the comparison between the GGA+U and the HSE06 results with standard α allows a good understanding of the effect of a Hubbard U vs. hybrid functional.

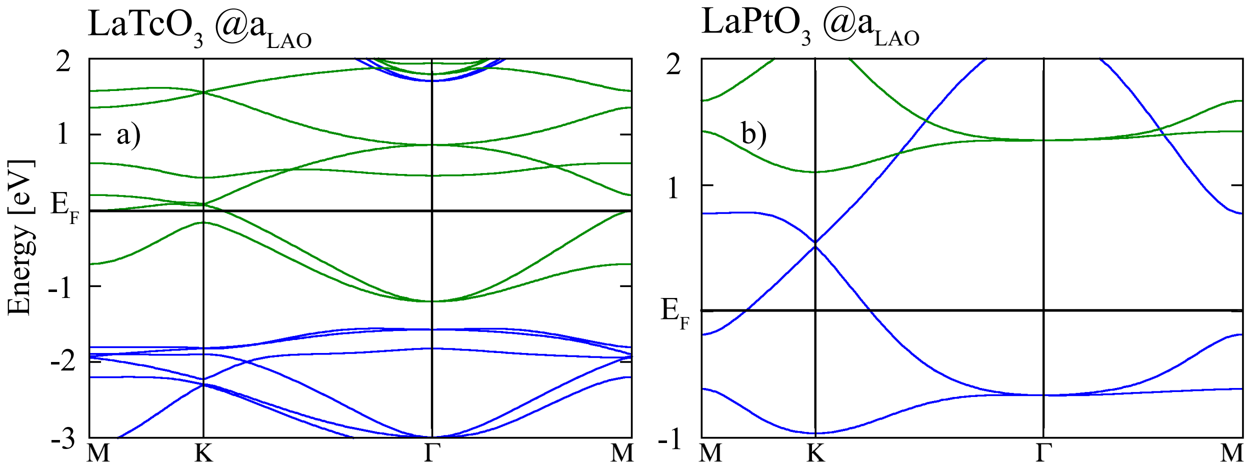

FIG. 1: Spin-resolved band structure of the buckled bilayers in (111)-oriented $(\mathrm{La}X\mathrm{O}_3)_2/(\mathrm{LaAlO}_3)_4$ perovskite bilayer (a-b) at a_{LAO} for $X = \mathrm{Tc}$ and Pt calculated within the hybrid functional HSE06, respectively. In the band structures blue/green denote majority/minority bands and the Fermi level is set to zero.

DYNAMIC STABILITY

We note that while the calculations of dynamical stability is typical for bulk materials it is uncommon for superlattices. We also point out that the growth of such superlattices is performed far from thermodynamic equilibrium which allows to realize systems that are otherwise unstable. Still we have addressed here the dynamical stability by carrying out Gamma phonon calculations for the ferromagnetic systems with constrained symmetry with $X = \mathrm{Tc}$ and Pt. Since the ground state in both cases is antiferromagnetic with lower symmetry, not surprisingly negative frequencies are found for the FM case with symmetric sublattices in the phonon density of states shown in Fig. 2a-b. Similar results were found previously for the corundum honeycomb layers $(X_2\mathrm{O}_3)_1/(\mathrm{Al}_2\mathrm{O}_3)_5$ (0001) with $X = \mathrm{Tc}$ and

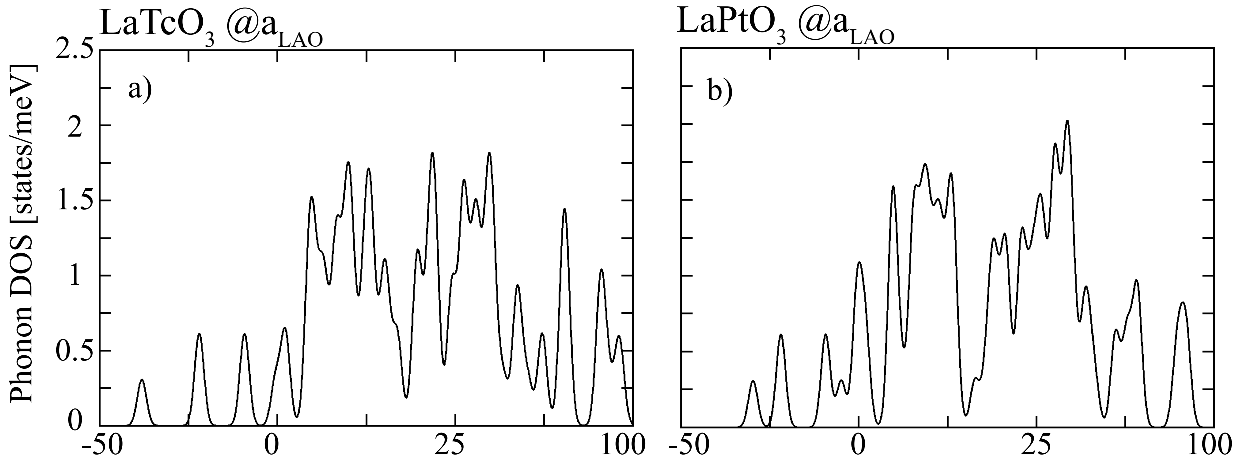

FIG. 2: Density of states (DOS) of Gamma phonons for$X = \mathrm{Tc}$ and Pt shown in a-b).

Pt [4] as well as $X = \mathrm{Ti}$ and Mn. On the other hand, as previously shown for the latter case, such systems can be stabilized by SOC [5]. However, due to their much higher computational demand, dynamic calculations with SOC go beyond the current scope.

* Electronic address: Rossitza.Pentcheva@uni-due.de

[1] J. Heyd, G. E. Scuseria, and M. Ernzerhof, J. Chem. Phys. **118**, 8207 (2003).
[2] J. Heyd, J. E. Peralta, G. E. Scuseria, and R. L. Martin, J. Chem. Phys. **123**, 174101 (2005).
[3] J. He and C. Franchini, Phys. Rev. B **86**, 235117 (2012)
[4] O. Köksal and R. Pentcheva, J. Phys. Chem. Solids **128**, 301-309 (2019).
[5] O. Köksal, S. Baidya, and R. Pentcheva, Phys. Rev. B **97**, 035126 (2018).
